# Field laboratory comparison of STANDARD Q Filariasis Antigen Test (QFAT) with Bioline Filariasis Test Strip (FTS) for the detection of Lymphatic Filariasis in Samoa, 2023

**DOI:** 10.1371/journal.pntd.0012386

**Published:** 2024-08-05

**Authors:** Jessica L. Scott, Helen J. Mayfield, Jane E. Sinclair, Beatris Mario Martin, Maddison Howlett, Ramona Muttucumaru, Kimberly Y. Won, Robert Thomsen, Satupaitea Viali, Rossana Tofaeono-Pifeleti, Patricia M. Graves, Colleen L. Lau

**Affiliations:** 1 College of Public Health, Medical and Veterinary Sciences, James Cook University, Douglas, Queensland, Australia; 2 School of Public Health, Faculty of Medicine, The University of Queensland, Brisbane, Queensland, Australia; 3 Centre for Clinical Research, Faculty of Medicine, The University of Queensland, Brisbane, Queensland, Australia; 4 School of Chemistry and Molecular Biosciences, The University of Queensland, Brisbane, Queensland, Australia; 5 National Centre for Epidemiology and Population Health, Australian National University, Canberra, Australian Capital Territory, Australia; 6 Division of Parasitic Diseases and Malaria, Centers for Disease Control and Prevention, Atlanta, Georgia, United States of America; 7 Samoa Ministry of Health, Apia, Samoa; 8 School of Medicine, National University of Samoa, Apia, Samoa; 9 Oceania University of Medicine Samoa, Apia, Samoa; Seoul National University College of Medicine, REPUBLIC OF KOREA

## Abstract

**Background:**

To monitor the progress of lymphatic filariasis (LF) elimination programmes, field surveys to assess filarial antigen (Ag) prevalence require access to reliable, user-friendly rapid diagnostic tests. We aimed to evaluate the performance of the new Q Filariasis Antigen Test (QFAT) with the currently recommended Filariasis Test Strip (FTS) for detecting the Ag of *Wuchereria bancrofti*, the causative agent of LF, under field laboratory conditions.

**Methodology/Principal findings:**

During an LF survey in Samoa, 344 finger-prick blood samples were tested using FTS and QFAT. Microfilariae (Mf) status was determined from blood slides prepared from any sample that reported Ag-positive by either Ag-test. Each test was re-read at 1 hour and the next day to determine the stability of results over time. Overall Ag-positivity by FTS was 29.0% and 30.2% by QFAT. Concordance between the two tests was 93.6% (kappa = 0.85). Of the 101 Mf slides available, 39.6% were Mf-positive, and all were Ag-positive by both tests. Darker test line intensities from Ag-positive FTS were found to predict Mf-positivity (compared to same/lighter line intensities). QFAT had significantly higher reported test result changes than FTS, mostly reported the next day, but fewer changes were reported between 10 minutes to 1hour. The field laboratory team preferred QFAT over FTS due to the smaller blood volume required, better usability, and easier readability.

**Conclusion/Significance:**

QFAT could be a suitable and user-friendly diagnostic alternative for use in the monitoring and surveillance of LF in field surveys based on its similar performance to FTS under field laboratory conditions.

## Author summary

Lymphatic filariasis (LF) is a debilitating tropical disease caused by infection with parasitic filarial worms that are transmitted by mosquitoes. Long-term infection can lead to stigmatising chronic conditions like lymphoedema and elephantiasis. The World Health Organization initiated the global programme to eliminate lymphatic filariasis (GPELF) in 2000, focusing on mass drug administration (MDA) of anti-LF drugs to stop transmission in endemic countries. However, to monitor the success of this programme and to make informed decisions to stop costly MDA, it is crucial to have access to accurate and reliable rapid diagnostics. Here, we evaluated the performance of a new rapid antigen test called the Q Filariasis antigen test and compared it to the currently recommended filariasis test strip under field laboratory conditions in Samoa. This study showed that the new rapid test could be a suitable alternative to the currently recommended test for use in GPELF-related activities with more user-friendly features.

## Introduction

Lymphatic filariasis (LF) is a mosquito-borne neglected tropical disease caused by infection with a parasitic worm. Consequences of long-term infection include chronic disabling and disfiguring manifestations such as lymphoedema, including scrotal hydrocele, and elephantiasis. These morbidities could lead to social stigmatisation and loss of work [1]. The main pathogen causing LF is the filarial worm *Wuchereria bancrofti* and, to a lesser extent, *Brugia malayi* and *B*. *timori* [2].

To eliminate LF as a public health problem, the World Health Organization (WHO) established the Global Programme to Eliminate Lymphatic Filariasis (GPELF) in 2000. One arm of the program is focused on interrupting the transmission of LF through repeated rounds of mass drug administration (MDA) of anti-filarial drugs in endemic regions [3]. In conjunction with the GPELF, the Pacific Programme to Eliminate LF (PacELF) was launched to support the 16 endemic Pacific Island Countries and Territories committed to combating this disease [4]. Since 1999, the elimination of LF as a public health problem has been validated in eight countries in the PacELF region, while the other eight, including Samoa, remain committed to eliminating the disease. Accessibility to robust and reliable diagnostic tools remains essential for monitoring the progress of GPELF activities and for post-validation surveillance [5].

Samoa has received multiple rounds of two-drug MDA since 1965 with accelerated efforts occurring upon the launch of the PacELF [6]. The MDA campaign that took place before this study was in 2019 when the triple-drug therapy was successfully implemented [7]. Since this time, other public health emergencies have taken precedence over conducting another MDA, halting LF elimination efforts. Renewed efforts are currently underway with the most recent MDA campaign conducted successfully in September 2023, after this study had taken place [8].

In regions where *W*. *bancrofti* is the leading causative agent of LF, WHO recommends the qualitative Alere Bioline Filariasis Test Strip (FTS; Alere Abbott), a rapid diagnostic test for detecting circulating filarial antigen in human blood samples. Since 2015, FTS has been successfully used in national programs, replacing the former immunochromatographic test (ICT, BinaxNOW). Previous studies have reported that FTS was preferred over ICT, as it was more stable in the field, cheaper, and able to detect lower concentrations of circulating filarial antigen than ICT [9], but its usability and user-friendliness under field conditions were considered a drawback as it is susceptible to user error [9,10]. Later, concerns were raised regarding potential cross-reactivity with other filarial species, such as *Loa loa* [11], although this parasite is not endemic in the Asia-Pacific. Further, since the COVID-19 pandemic, relying on a single manufacturer has introduced procurement challenges for rapid diagnostic tests, which has been a barrier for LF programmatic surveys.

A new rapid antigen test, the Q Filariasis Antigen test (QFAT) (SD Biosensor, Suwon, South Korea), has been proposed as an alternative to the FTS for GPELF-related LF surveys. This new test also detects filarial antigens from capillary blood, like FTS and requires less sample volume, but its utility under field laboratory conditions has not been evaluated. A previous laboratory study evaluated the performance of QFAT using a panel of retrospectively collected and tested blood samples from LF endemic regions in the Asia-Pacific [12]. It was revealed in this study that QFAT reacts with *W*. *bancroft*i detected in Asia-Pacific with high specificity (98%), and has a comparable, albeit slightly higher sensitivity than FTS [12].

In this current study, we aimed to evaluate the performance of the new QFAT and FTS when deployed in an endemic region under field laboratory conditions. The specific objectives were to compare the concordance between the two tests for detecting the circulating antigen of *W*. *bancrofti*, to assess whether the intensity of the test line of QFAT could indicate microfilariae positivity and determine test stability over time by re-reading the tests at one hour and the next day. The findings from this study will support recommendations regarding the suitability of QFAT as an alternative field diagnostic for GPELF-related activities.

## Materials and methods

### Ethical approval statement

Ethics approvals were obtained from the Samoa Ministry of Health and the Human Research Ethics Committee (HREC) of The University of Queensland (protocol 2021/HE000895) and the Australian National University HREC provided recognition of approval by another HREC. Formal written consent was obtained for all participants. Further, children participating in this study had formal written consent obtained from the parent/guardian.

### Study setting and participants

Field and laboratory work for this study was undertaken in Samoa, on the main island of Upolu, in 2023. All the samples used in this study were collected in 2023 and are associated with three study components that were part of the monitoring and surveillance field project for LF in Samoa. The first study component included participants who were followed up (and their household members) from surveys conducted in Samoa in 2018 and 2019 [7,13], who tested antigen (Ag) positive by FTS and had detectable microfilaria (Mf) in blood, as identified by microscopy. The second component included participants from a community-based survey of eight sentinel villages, which were selected based on Ag prevalence reported in 2019 (two villages, each with zero, low (3–4%), medium (6–7%) and high (13–16%) prevalence). The final component included participants who were targeted for testing based on Ag-positivity of residents in neighbouring households identified in a 2019 survey.

### Blood sample collection and processing

Heparinised microvettes were used to collect 300μL of capillary (finger-prick) blood samples from participants. Ambient temperatures at the time of collection were 25–32°*C*. Immediately after collection, blood samples were stored in a cold storage container for no longer than 6 hours until the samples were delivered to the field laboratory and stored in the fridge at 4°C. Typically, samples were processed and tested the next morning, or two days later if collected on Saturdays.

Before testing, samples were acclimated to room temperature and tested in a field laboratory. The rapid tests were performed per the manufacturer’s instructions using 75 μL of blood for FTS [14] and 20 μL for QFAT [15]. The precise volumes were transferred to the respective tests using a calibrated micropipette instead of the supplied capillary tubes. A comparison of QFAT and FTS test characteristics are summarised in S1 Table.

### Selection of blood samples for QFAT comparison

To select the samples for the QFAT trial, all samples from the wider survey were tested with FTS in batches of five. Any batch of five samples that included at least one FTS-positive sample was also tested using QFAT. This strategy ensured sufficient numbers of Ag-positive samples for comparisons between FTS and QFAT. Therefore, it is important to note that the Ag positivity rate presented in this study does not represent the prevalence in any of the three study components described above. In addition to the above selection strategy, 14 samples that were inadvertently frozen and invalid by FTS were purposefully included in the QFAT trial for comparison.

### Reading and interpretation of rapid antigen test results

Results of FTS and QFAT were read at the manufacturer’s recommended time of 10 minutes by up to three independent and blinded readers. Occasionally, a high laboratory workload meant that it was not possible for all tests to be read by three readers during the short time window for reading. Tests were read by the naked eye and illuminated with a torch if needed. Results were classified by each reader as positive, negative, or invalid (no sample flow or absence of control line). Samples with invalid results were repeated if there was sufficient blood. If the second test produced a valid result, this was recorded as the final result. If the second test produced another invalid result, it was recorded as invalid.

At the 10-minute readings, tests with a positive result had the test line semi-quantified based on the intensity of the test line compared to the control line. These tests were scored by up to three readers, accordingly: (1) test lines were lighter than the control line; (2) test lines were the same intensity as the control line; or (3) test lines were darker than the control line. Each test was then re-read at 1 hour and the next day (e.g. 12–18 hours) by one reader to determine whether the tests remained stable over time. Re-reading the Ag tests after 10 minutes is not recommended by the manufacturers.

### Preparation of thick smear blood films for the determination of Mf status

Blood samples testing positive for LF Ag by FTS and/or QFAT, up to three thick smear slides were prepared (depending on the blood volume available). Each slide included three 20 μL stripes of blood. Slides were dried, dehaemoglobinised in water and stained with Giemsa following standard procedures [16].

### Data analysis

Data was analysed using GraphPad Prism 9 (Prism for Windows, version 9.2.0.332). FTS and QFAT summaries were described as frequencies and percentages and reported with a 95% confidence interval (CI). Final interpretations of the test results for FTS and QFAT were determined by the consensus between the readers and were done accordingly; tests with discordant readings between readers, the dominant reading was used as the result. If the readings were discordant for tests with only two readers, it was classified as indeterminant. In cases where a single reader provided the interpretation, this reading was considered final. Discordance between the readers was determined when test interpretations made by the readers differed from one another and the analysis excluded samples with invalid interpretations. This consensus-based approach was similarly applied to the semi-quantification scores determined for both Ag-tests. However, tests with only two readers who provided discordant semi-quantification scores were excluded from the analysis.

Concordance between the results of the two rapid Ag tests (agreement or disagreement between positive and negative results) was determined and reported as a percentage. Cohen’s Kappa (Κ) agreement statistics were performed to determine the probability that agreement between the tests was not due to chance and reported with a 95% CI. Line intensity scores from Ag-positive tests to use for the analysis were selected based on the consensus of test line intensity scores by the independent readers. If no consensus was reached by the readers for a particular test result, the result was excluded. These line intensity scores for FTS and QFAT were then compared for those samples where Mf-status was determined. A univariable logistics regression analysis was conducted to assess whether the darker test lines of Ag-positive tests (compared to those with equal or lesser intensity than the control line) were predictive of Mf-positivity. The relationship was presented as an odds ratio (OR) and reported with a 95% CI.

FTS and QFAT tests were re-read at 1 hour and next day time points to determine whether the test results changed over time (from Ag-negative to Ag-positive, or vice versa). Tests with valid positive or negative results at all three time points were compared. The McNemar test was used to determine the difference between the proportion of tests that changed results from 10 minutes to 1 hour, and from 1 hour to the next day. Fisher’s exact test was used to determine the difference between the proportion of tests that changed results for FTS and QFAT at 1 hour and next day readings. All statistical inferences were based on a p-value of <0.05.

Mf-status was determined by two independent blinded readers who examined at least two slides per participant. If at least one slide reader detected any Mf, it was classified as Mf-positive. However, this discordance of Mf results between the two slide readers was validated by jointly re-examining the slides and making a final judgment. If neither slide reader detected Mf, it was classified as Mf-negative.

### QFAT usability under field laboratory conditions

Four field laboratory workers, trained before sample testing, provided independent verbal feedback on FTS and QFAT at the conclusion of the field laboratory comparison. Two of the four laboratory workers have had previous experience using FTS and/or QFAT. Laboratory workers were asked for feedback on test set-up, sample application and volume, test readability and other relevant aspects of the test’s characteristics and procedure. Prompt questions for the semi-structured interview are supplied in S1 Questionnaire.

## Results

A total of 344 whole blood samples were tested using both FTS and QFAT and were used to evaluate test concordance at the initial reading (10 minutes after application of the sample). The median age of participants included in this study was 26 years (range: 5–87 years) and 55% were female. At the 10minute reading, no indeterminant results were reported for QFAT, but three (1.0%) were reported for FTS. Of the 14 (4.1%) samples that were invalid by FTS, none were reported as invalid by QFAT. For the invalid FTS readings, no control lines were reported for the tests, even after repeating the samples on a newer batch of test strips. All 14 samples invalid by FTS produced a valid result by QFAT (one positive and thirteen negatives). Thick blood smears were prepared for 101 Ag-positive participants, and 40 (39.6%, 95% CI 30.6–49.4%) of these were classified as Mf-positive. After the initial reading, 309 (89.8%) of the FTS and 341 (99.1%) of QFAT were read again at 1 hour and the next day and were used for further analysis.

### Number of readers and discordance between readers at initial reading

Among the 344 samples evaluated and removal of the 14 invalid FTS interpretations, 70.3% of FTS and 84.6% of QFAT were read by three independent blinded readers. The total proportion of interpretation discordance between readers was highest for QFAT (4.4%) than FTS (2.1%). Specifically, for tests with only two readers, there was higher discordance for FTS (1.0%) than QFAT (0%), but for tests with three readers, discordance for QFAT (4.4%) was higher than FTS (1.2%). The number of readers for FTS and QFAT at the initial reading (10 minutes) and discordance between the readers is summarised in S2 Table.

### LF antigen positivity by FTS and QFAT

Overall, 100 samples tested positive with FTS (29.0%, 95% CI 24.5–34.1%) and 104 samples with QFAT (30.2%, 95% CI 25.6–35.3%). If indeterminant and invalid FTS results were excluded from the analysis, Ag positivity was 30.6% (95% CI 25.8–35.8%) by FTS and 30.2% (95% CI 25.6–35.3%) by QFAT.

### FTS and QFAT concordance at initial reading (at 10 minutes)

The overall concordance between FTS and QFAT, including indeterminant and invalid readings (n = 344), was 93.6%. The kappa agreement statistic indicated excellent agreement between the two tests (Κ = 0.85; 95% CI 0.80–0.91). Table 1 demonstrates that of the valid but discordant test results, one was positive by FTS and negative by QFAT, and four were positive by QFAT and negative by FTS.

**Table 1:** Concordance between Filariasis Test Strip and Q Filariasis Antigen Test results, including indeterminant and invalid tests at 10minutes, Samoa 2023.

**QFAT**		**FTS**					**Concordance (%)**	**Kappa** **(95% CI)**
		**Positive**	**Negative**	**Indeterminant**	**Invalid**	**Total**		
	**Positive**	99	4	0	1	**104**	93.6	0.85 (0.80–0.91)
	**Negative**	1	223	3	13	**240**		
	**Indeterminant**	0	0	0	0	**0**		
	**Invalid**	0	0	0	0	**0**		
	**Total**	**100**	**227**	**3**	**14**	**344**		

CI: Confidence interval; FTS: Filariasis Test Strip; QFAT: Q Filariasis Antigen Test

If indeterminant and invalid readings were excluded from the analysis, the concordance between FTS and QFAT (n = 327) improved to 98.5% (Table 2). The kappa agreement similarly indicated excellent agreement between the two tests (Κ = 0.96; 95% CI 0.96–1.0).

**Table 2:** Concordance between Filariasis Test Strip and Q Filariasis Antigen Test for all samples and stratified by microfilaria positive or negative, excluding indeterminant and invalid results at 10minutes, Samoa 2023.

Samples with valid results for FTS and QFAT	N	QFAT	FTS		Concordance (%)	Kappa (95% CI)
			Positive	Negative		
**All samples**	327	Positive	99	4	98.5	0.96 (0.96–1.0)
		Negative	1	223		
**Mf-positive**	40	Positive	40	0	100.0	NA
		Negative	0	0		
**Mf-negative**	61	Positive	57	3^‡^	93.4	NA
		Negative	1	0		

Mf: Microfilaria; CI: Confidence interval; FTS: Filariasis Test Strip; QFAT: Q Filariasis Antigen Test. NA: Not applicable.

^‡^ One Ag-positive individual had an uninterpretable slide

All 40 Mf-positive samples were Ag-positive by both FTS and QFAT. Of the 61 Mf-negative samples, 57 were Ag-positive by both QFAT and FTS, with 93.4% concordance between the two tests (Table 2).

### Antigen-positive FTS and QFAT test line intensity scores

**Fig 1:**
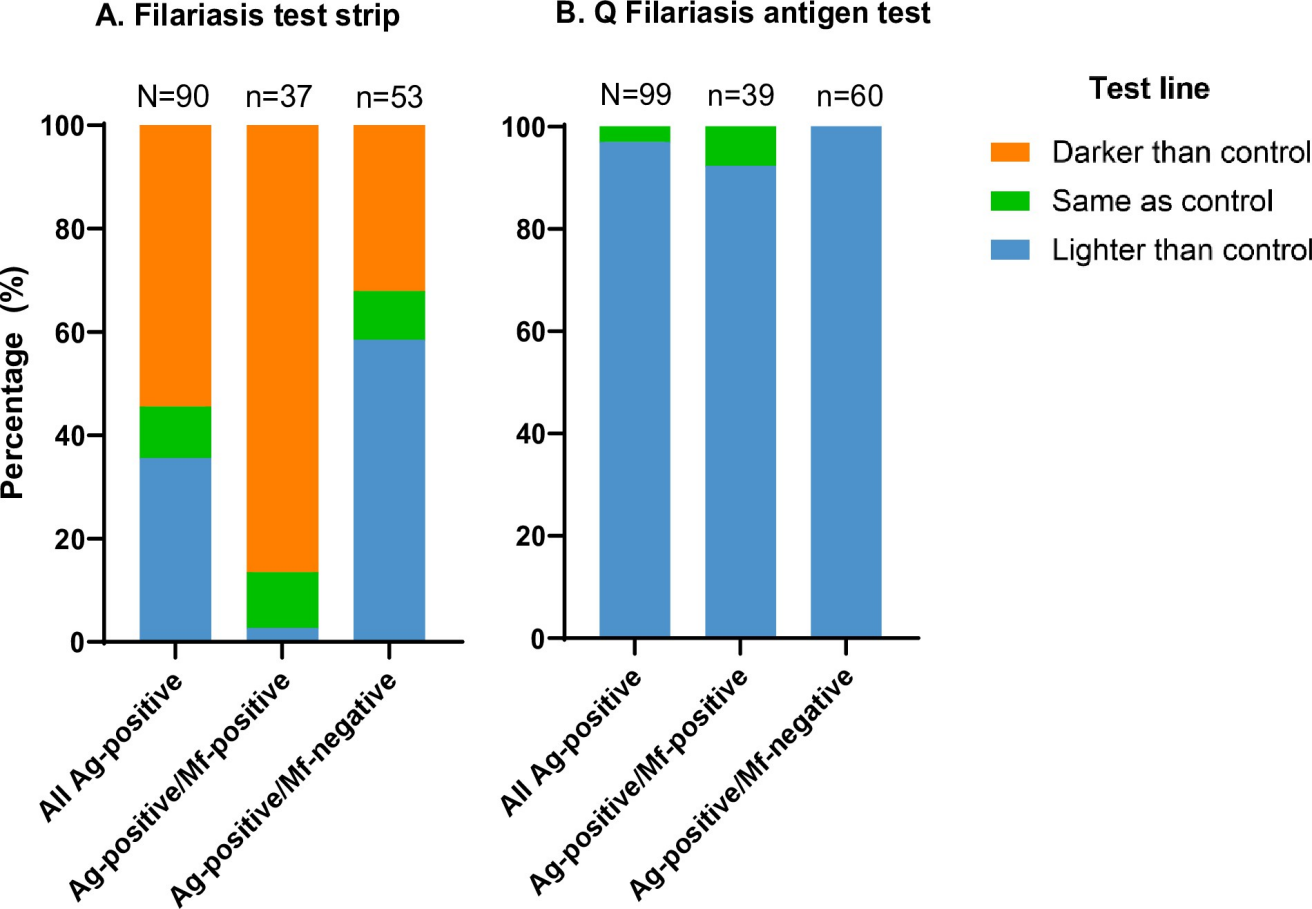
Summary of semi-quantitative scoring of antigen tests. Semi-quantitative scoring (darker, same or lighter than the control line) determined for participants with antigen (Ag)-positive (A) filariasis test strip and (B) Q Filariasis antigen tests, against microfilariae (Mf) status.

The distribution of the FTS and QFAT test line intensity scores among the Mf-positive and Mf-negative participants are presented in Fig 1. Blood samples that were Ag-positive by FTS and Mf-positive produced predominantly strong (3) test lines (86.5%), compared to FTS Ag-positive/Mf-negative samples. In contrast, blood samples testing Ag-positive by QFAT and Mf-positive had mostly light (1) intensity test lines (92.4%), similar to test line intensities reported for QFAT Ag-positive and Mf-negative blood samples. For samples that tested positive for Ag by FTS, those with test lines darker than the control had 13.6 times higher odds of being Mf-positive (95% CI 4.8–45.4), compared to those with test lines that were the same or lighter intensity than the control (p-value <0.0001). However, a logistic regression could not be applied for QFAT because there were no samples with darker test lines than the control line.

### Changes in FTS and QFAT test results at 1 hour and next day timepoints

We assessed the difference between FTS and QFAT test results at three time points (10minutes, 1 hour and next day) to determine whether test results remained stable over time. Only data sets with complete and valid readings across all three-time points were included in the analysis. Data excluded from the analysis were from the 1hour timepoint due to missing data. Ag positivity at 10 minutes was 30.1% by FTS and 29.9% by QFAT. At 1 hour, Ag positivity was 30.2% by FTS and 29.6% by QFAT. The next day, the Ag positivity remained at 30.2% for FTS but increased to 39.6% for QFAT.

A total of 46 (13.5%) QFAT tests were observed to have results change between 10 minutes and 1 hour and/or the next day, while five (1.6%) changed for FTS. A smaller proportion of QFAT (0.9%) tests compared to FTS (1.6%) were reported to have changed in result interpretations from 10 minutes to 1 hour. The difference between these observations was not statistically significant. The proportion of QFAT tests that had a reported change of results between the 1 hour and next day readings was statistically significant (McNemar test p-value = 0.003). Additionally, there was a statistically significant difference between the proportion of tests changing for FTS and QFAT at the next-day readings (Fisher’s exact test p-value = 0.006). These results indicate minor changes in test result readings for FTS and QFAT between 10 minutes and 1 hour, whereas, at the next-day readings, the number of test results reported to have changed was significantly greater for QFAT than for FTS. The distinct proportion of tests reported to have changed at these time points for FTS and QFAT have been described in S3 Table and S1 Fig.

### QFAT usability under field laboratory conditions

The field laboratory team reported that QFAT was preferred over FTS for multiple reasons. QFAT required smaller sample volumes than FTS. Further, the smaller sample volume required for QFAT made it easier to apply the blood sample, as blood spillage off the application pad was occasionally noted with FTS but not QFAT. The QFAT cassette was easier to handle than the loose strip and tray used for the FTS, and QFAT occupied less space in the field laboratory. Furthermore, the control line for QFAT was consistently clearer and easier to identify than for FTS. Although the test line on QFAT sometimes appeared lighter than the control, it was still identifiable even without a torch, unlike FTS, which could be challenging to see at times. It was also noted that in times of high laboratory demand, the additional buffer step required for QFAT could be missed, which could potentially lead to invalid test results.

## Discussion

Our study found an excellent level of agreement between FTS and QFAT for LF Ag results. Ag positivity based on initial 10minute readings was similar for both tests, with a slightly higher reported positivity rate for QFAT. This finding is similar to the laboratory evaluation of QFAT reported in a recent study [12]. Additionally, it was encouraging to find that all Mf-positive samples were Ag-positive by both QFAT and FTS as these samples tend to have higher levels of detectable circulating Ag [17]. Discordance between the independent readers was highest for QFAT compared to FTS. However, all tests were resolvable due to the consensus among readers, except for 1.0% of FTS test results that were classified as indeterminant.

Additionally, we found that darker test line intensities, compared to the control line, produced by Ag-positive FTS was a predictor of Mf-positivity. However, this was not the case for QFAT, as no samples produced a test line that was darker than the control line. Indicating that the semi-quantification of the test line for Ag-positive QFAT tests may have limited utility for indicating Mf-positivity. It has been previously suggested that semi-quantification of the test line as compared to the control line could indicate Mf-positivity from blood samples [18], which could have potential utility in field studies to indirectly infer the level of Mf rates in a population over time [19]. However, the findings from this current study could be influenced by the smaller sample volume required for QFAT than for FTS, as the greater intensity of the FTS test line noted in this study could be influenced by the test’s greater sample volume requirement. It should also be noted that QFAT consistently had stronger control lines as compared to FTS, whereby the control line of FTS was often very faint. The darker control lines are more reliable as they are less prone to misinterpretations and being discarded as invalid.

A significantly greater proportion of tests were reported to have changed for QFAT than FTS at the next day readings. The propensity of the reported change was negative to positive. We found that only a small proportion of tests had a reported change in test interpretations from 10 minutes to 1 hour. However, past this 1hour time point, it was found that the results interpreted the next day could be unreliable. A previous study comparing and assessing the stability of the former ICT with FTS over time revealed that participants from a non-endemic country had Ag-negative FTS tests at 10 minutes, but after 24 hours, some of the tests turned positive [9], indicating some level of unreliability of FTS if test results are read after the recommended time. Although the findings from this study suggest that test results produced by QFAT could be read up to 1hour post application of blood, further studies with larger sample sizes will be needed to validate this finding.

In general, the field laboratory team preferred QFAT over FTS predominantly due to its smaller sample volume requirements, which would be beneficial to field workers as it allows sufficient volumes to be used for other purposes (e.g., Mf slides and/or dried blood spots) or for repeating FTS or QFAT if needed, but also for its user-friendliness. The field team also reported that QFAT was easier to use and interpret the results. However, the evaluation of user-friendliness may be limited as micropipettes were used to apply precise volumes to the test sample pad, deviating from the capillary tubes provided by the test kits.

Reliability and user-friendliness of diagnostic tests are key criteria of the target product profiles (TPP) outlined by the WHO’s Diagnostics Technical Advisory Group (DTAG) for NTDs to be fulfilled when seeking recommendations for test implementation into field surveys [20]. Our findings suggest that the test format of QFAT improves upon the user-friendly shortcomings of FTS [10].

The strengths of this study include the ability to perform QFAT under field laboratory conditions, given its integration within an LF community survey and alongside FTS testing. However, in LF programmes, QFAT is intended for point-of-care application, specifically using blood directly from a finger prick. This approach was not evaluated in this current study. While QFAT has demonstrated promising performance under field laboratory conditions in Samoa, additional field evaluations are recommended for other settings and should consider evaluating the point-of-care aspect. No major differences in test performance would be expected between point-of-care testing versus testing of the blood samples in the improvised field laboratory setting in this study. However, the use of capillary tubes as opposed to micropipettes might impact the usability of the test by field staff. Extra considerations should also be made as to whether temporary storage and anti-coagulation of the blood samples could influence test results and interpretations.

Additionally, having multiple readings of the test results at the initial 10 minutes was an advantage to this study. Multiple readers enabled the resolution of discordant results, enhancing the reliability of the conclusions drawn from this study. Cross-reactivity was not explored in this current study and while concerns of cross-reactivity of FTS with human *L*. *loa* [11] and potentially *Strongyloides* spp. infections [9] have been raised, it is unlikely an issue for QFAT [12]. Supporting this, the laboratory study that evaluated the specificity of QFAT found some evidence of cross-reactivity with two canine filarial worms (*Dirofilaria repens* and *Onchocerca lupi*), but not with human *Strongyloides* spp. [12]. Although the sample size in the current study was limited by the number of QFAT cassettes available, our robust statistical methods demonstrated a high level of agreement between QFAT and FTS, with kappa of 0.85 for all samples and 0.96 if indeterminant and invalid readings were excluded. Additionally, due to limited resources, blood smears to detect Mf in participants with Ag-negative results by QFAT and FTS were not prepared. However, the likelihood of someone who is Ag-negative being Mf-positive is low [21].

Remaining knowledge gaps include whether QFAT will produce comparable results to FTS after repeated rounds of MDA, Ag prevalence is low, and whether test accuracy varies by age group and biological sex, as previously shown for FTS [22]. Additionally, a cost-benefit analysis would be valuable to determine if QFAT’s accuracy and user-friendliness demonstrated in this study lead to cost savings compared to using FTS.

In summary, QFAT demonstrated promising performance under field laboratory conditions in Samoa. Reliability and user-friendliness are key TPP criteria outlined by DTAG-NTDs. Our findings show that QFAT is reliable, as Ag-positivity rates were comparable to the currently recommended FTS, with excellent concordance found between the two tests. Additionally, the field laboratory team preferred QFAT over FTS due to its smaller sample volume requirements, ease of use, and clearer readability. These results suggest that QFAT is a reliable and user-friendly Ag detection test that could be a suitable alternative in LF surveillance and control programs.

## Supporting information

S1 Table Comparing test characteristics for the FTS and QFAT. (DOCX)

S2 Table Number of readers for Filariasis Test Strip and Q Filariasis Antigen Test at initial reading (at 10 minutes) and discordant results between readers, excluding invalid test interpretations, Samoa 2023. (DOCX)

S3 Table Antigen positivity at the time intervals, 10 minutes, 1 hour and the next day. Total sample size included for this analysis was 300 for FTS and 341 for QFAT.
(DOCX)

S1 Questionnaire Semi-structured prompt questions for the field laboratory staff interview on the usability of QFAT. (DOCX)

S1 Fig Changes in readings for FTS (A) and QFAT (B) from the initial 10-minute reading to 1 hour and next-day readings post application of blood. (DOCX)
