# Supplementary material for: Field laboratory comparison of STANDARD Q Filariasis Antigen Test (QFAT) with Bioline Filariasis Test Strip (FTS) for the detection of Lymphatic Filariasis in Samoa, 2023
Source: PLoS Negl Trop Dis. 2024 Aug 5;18(8):e0012386. doi: 10.1371/journal.pntd.0012386 (PMC11326698; doi:10.1371/journal.pntd.0012386)
Supplement: S1 Table — (DOCX) [file pntd.0012386.s001.docx]

**Field laboratory comparison of STANDARD Q Filariasis Antigen Test (QFAT) with Bioline Filariasis Test Strip (FTS) for the detection of Lymphatic Filariasis in Samoa, 2023**

Jessica L Scott, Helen J Mayfield, Jane E Sinclair, Beatris Mario Martin, Maddison Howlett, Ramona Muttucumaru, Kimberly Y Won, Robert Thomsen, Satupaitea Viali, Rossana Tofaeono-Pifeleti, Patricia M Graves, Colleen L Lau

**S1 Table. Comparing test characteristics for the FTS and QFAT**

|  | **Filariasis Test Strip**  **(Alere Abbott)** | **Q Filariasis Antigen Test**  **(SD Biosensor)** |
| --- | --- | --- |
| **Sample volume:** Whole blood | 75$\mu$L | 20$\mu$L |
| **Time until result** | 10 minutes | 10 minutes |
| **Buffer** | No | Yes |
| **Test format** | Single test strip, devoid of protective housing with plastic tray and adhesive tape to secure test strip. | Plastic protective housing of the test. |


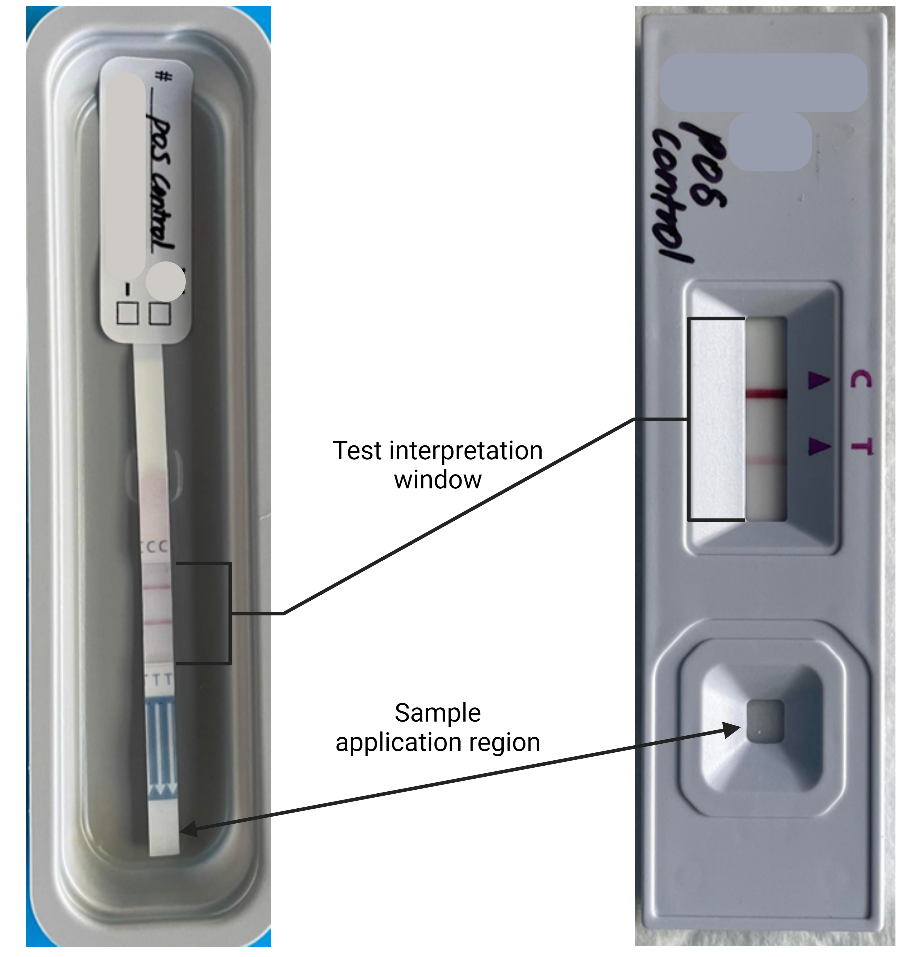
Pos; positive

Filariasis Test Strip (left) and Q Filariasis Antigen Test (right). Photos captured by author JLS (2023).
