# Supplementary material for: Field laboratory comparison of STANDARD Q Filariasis Antigen Test (QFAT) with Bioline Filariasis Test Strip (FTS) for the detection of Lymphatic Filariasis in Samoa, 2023
Source: PLoS Negl Trop Dis. 2024 Aug 5;18(8):e0012386. doi: 10.1371/journal.pntd.0012386 (PMC11326698; doi:10.1371/journal.pntd.0012386)
Supplement: S2 Table — (DOCX) [file pntd.0012386.s002.docx]

Field laboratory comparison of STANDARD Q Filariasis Antigen Test (QFAT) with Bioline Filariasis Test Strip (FTS) for the detection of Lymphatic Filariasis in Samoa, 2023

Jessica L Scott, Helen J Mayfield, Jane E Sinclair, Beatris Mario Martin, Maddison Howlett, Ramona Muttucumaru, Kimberly Y Won, Robert Thomsen, Satupaitea Viali, Rossana Tofaeono-Pifeleti, Patricia M Graves, Colleen L Lau

S2 Table. Number of readers for FTS and QFAT at initial reading (at 10 minutes) and discordant results between readers, excluding invalid test interpretations, Samoa 2023.

|  | **FTS** | | **QFAT** | |
| --- | --- | --- | --- | --- |
| **Number of readers** | **Total number of samples (%)** | **Discordant interpretations from the total number of samples (%)** | **Total number of samples (%)** | **Discordant interpretations from the total number of samples (%)** |
| **3** | 232 (70.3%) | 4 (1.2%) | 291 (84.6%) | 15 (4.4%) |
| **2** | 80 (24.2%) | 3 (1.0%) | 48 (13.9%) | 0 (0%) |
| **1** | 18 (5.5%) | - | 5 (1.5%) | - |
| **Overall** | 330 (100%) | 7 (2.1%) | 344 (100%) | 15 (4.4%) |
