# Supplementary material for: Field laboratory comparison of STANDARD Q Filariasis Antigen Test (QFAT) with Bioline Filariasis Test Strip (FTS) for the detection of Lymphatic Filariasis in Samoa, 2023
Source: PLoS Negl Trop Dis. 2024 Aug 5;18(8):e0012386. doi: 10.1371/journal.pntd.0012386 (PMC11326698; doi:10.1371/journal.pntd.0012386)
Supplement: S3 Table — Total sample size included for this analysis was 300 for FTS and 341 for QFAT. (DOCX) [file pntd.0012386.s003.docx]

Field laboratory comparison of STANDARD Q Filariasis Antigen Test (QFAT) with Bioline Filariasis Test Strip (FTS) for the detection of Lymphatic Filariasis in Samoa, 2023

Jessica L Scott, Helen J Mayfield, Jane E Sinclair, Beatris Mario Martin, Maddison Howlett, Ramona Muttucumaru, Kimberly Y Won, Robert Thomsen, Satupaitea Viali, Rossana Tofaeono-Pifeleti, Patricia M Graves, Colleen L Lau

S3 Table. Antigen positivity at the time intervals, 10 minutes, 1 hour and the next day. Total sample size included for this analysis was 300 for FTS and 341 for QFAT.

| **Timepoints** | **FTS** | | **QFAT** | |
| --- | --- | --- | --- | --- |
|  | **Number of tests positive** | **% (95% CI)** | **Number of tests positive** | **% (95% CI)** |
| **10 minutes** | 93 | 30.1 (25.3-35.4) | 102 | 29.9 (25.3-35.0) |
| **1 hour** | 94 | 30.2 (25.4-35.5) | 101 | 29.6 (25.0-34.7) |
| **Next day** | 94 | 30.2 (25.4-35.5) | 135 | 39.6 (34.5-44.9) |

CI: 95% confidence interval; FTS: Filariasis Test Strip; QFAT: Q Filariasis Antigen Test
