# Supplementary material for: Field laboratory comparison of STANDARD Q Filariasis Antigen Test (QFAT) with Bioline Filariasis Test Strip (FTS) for the detection of Lymphatic Filariasis in Samoa, 2023
Source: PLoS Negl Trop Dis. 2024 Aug 5;18(8):e0012386. doi: 10.1371/journal.pntd.0012386 (PMC11326698; doi:10.1371/journal.pntd.0012386)
Supplement: S1 Questionnaire — (DOCX) [file pntd.0012386.s004.docx]

**Field laboratory comparison of STANDARD Q Filariasis Antigen Test (QFAT) with Bioline Filariasis Test Strip (FTS) for the detection of Lymphatic Filariasis in Samoa, 2023**

Jessica L Scott, Helen J Mayfield, Jane E Sinclair, Beatris Mario Martin, Maddison Howlett, Ramona Muttucumaru, Kimberly Y Won, Robert Thomsen, Satupaitea Viali, Rossana Tofaeono-Pifeleti, Patricia M Graves, Colleen L Lau

**S1 Questionnaire. Semi-structured prompt questions for the field laboratory staff interview on the usability of QFAT.**

1. How did you find the clarity of the instructions? Were there any parts that were confusing or difficult to follow?
2. Can you describe your experience with the packaging of the test? Was it easy to open and access the contents?
3. What are your thoughts on the overall setup of the test? Were there any specific aspects you found particularly well-designed or problematic?
4. How did you find the process of applying the sample? Were there any difficulties or issues you encountered?
5. How easy was it to interpret the control line on the test? Did you have any trouble understanding what it indicated?
6. How easy was it to interpret the test line? Was it clear what the results meant?
7. Do you have any additional comments or suggestions regarding the test?
