# Supplementary material for: Field laboratory comparison of STANDARD Q Filariasis Antigen Test (QFAT) with Bioline Filariasis Test Strip (FTS) for the detection of Lymphatic Filariasis in Samoa, 2023
Source: PLoS Negl Trop Dis. 2024 Aug 5;18(8):e0012386. doi: 10.1371/journal.pntd.0012386 (PMC11326698; doi:10.1371/journal.pntd.0012386)
Supplement: S1 Fig — (DOCX) [file pntd.0012386.s005.docx]

Field laboratory comparison of STANDARD Q Filariasis Antigen Test (QFAT) with Bioline Filariasis Test Strip (FTS) for the detection of Lymphatic Filariasis in Samoa, 2023

Jessica L Scott, Helen J Mayfield, Jane E Sinclair, Beatris Mario Martin, Maddison Howlett, Ramona Muttucumaru, Kimberly Y Won, Robert Thomsen, Satupaitea Viali, Rossana Tofaeono-Pifeleti, Patricia M Graves, Colleen L Lau

S1 Fig. Changes in readings for FTS (A) and QFAT (B) from the initial 10-minute reading to 1 hour and next-day readings post application of blood.

**10 minutes**

**1 hour**

**Next day**


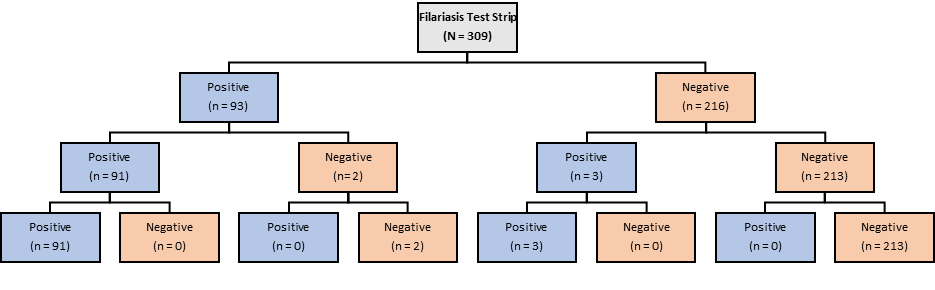


**A**

**10 minutes**

**1 hour**

**Next day**


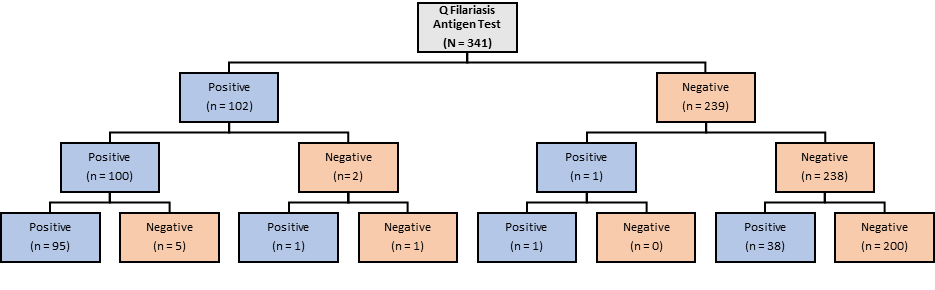


**B**
